# What Is the Response Seen During Para-Hisian Pacing?

**DOI:** 10.19102/icrm.2021.120905

**Published:** 2021-09-15

**Authors:** Khalil Kanjwal, Muzaffar Ali, John Catanzaro, Binu Philips, Sameer Jamal, Takeki Suzuki, Sang yong Ji, Sandeep Bansal

**Affiliations:** ^1^Section of Electrophysiology McLaren Greater Lansing, Michigan State University, Lansing, MI, USA; ^2^Division of Cardiology Sheri-Kashmir Institute of Medical Sciences, Kashmir, India; ^3^Division of Cardiology, University of Florida—Jacksonville, Jacksonville, FL, USA; ^4^Mount Auburn Hospital, Cambridge, MA, USA; ^5^Section of Electrophysiology, Hackensack University Medical Center, Hackensack, NJ, USA; ^6^Division of Cardiology, Krannert Institute of Cardiology, School of Medicine, Indiana University, Indianapolis, IN, USA; ^7^Division of Cardiac Electrophysiology, Torrance Memorial Medical Center, Torrance, CA, USA; ^8^Department of Cardiology, Lancaster General Hospital, Lancaster, PA, USA

**Keywords:** Nodal response, para-Hisian pacing, supraventricular tachycardia

## Abstract

We present an interesting tracing of para-Hisian pacing in a 45-year-old man with an episode of narrow complex tachycardia and past recurrent palpitations.

## Case presentation

**Figure 1:**
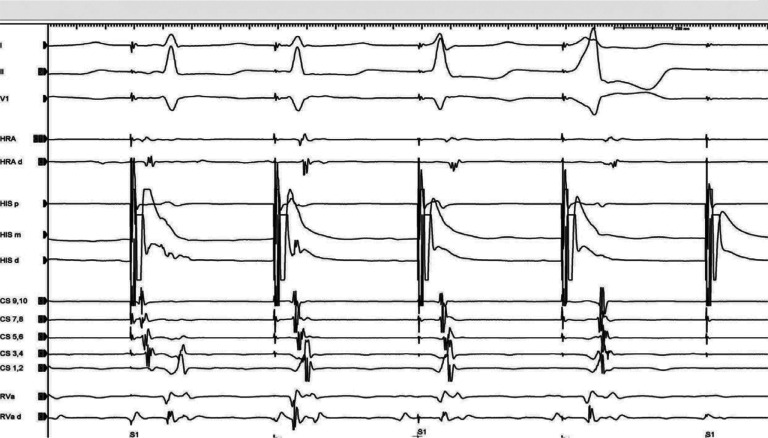
Para-Hisian pacing during sinus rhythm. The first beat is a direct atrial capture, the second beat is a His capture, and the third beat is both His and ventricular capture. The fourth beat is a ventricular capture and the fifth beat is a non-capture. CS: coronary sinus; d: distal; HRA: high right atrium; m: middle; p: proximal; RVa: right ventricular apex.

A 45-year-old man with a history of recurrent palpitations and an episode of narrow complex tachycardia was sent to our arrhythmia clinic for further evaluation. Given the multiple episodes of palpitations and multiple emergency room visits, the patient was offered an electrophysiology (EP) study with possible ablation and was subsequently taken to the EP lab. During the EP study, para-Hisian pacing was performed **(Figure 1)** during sinus rhythm. The responses to para-Hisian pacing are demonstrated in **Figure 1**. We herein consider the best explanation for the responses in this figure.

## Discussion

Para-Hisian pacing is performed during sinus rhythm, and this maneuver helps to distinguish whether midline retrograde conduction occurs through the atrioventricular (AV) node or a septal accessory pathway. Pacing is performed either from the His catheter or preferably through a separate catheter placed close to the His bundle. Pacing is started at a high output to capture the His bundle directly and the surrounding myocardial tissue. The pacing output is gradually decreased until His capture is lost. When the His is captured, the resulting QRS will be narrow, and when His capture is lost, the QRS will widen into a left bundle branch block–like pattern.^1^

**Figure 2:**
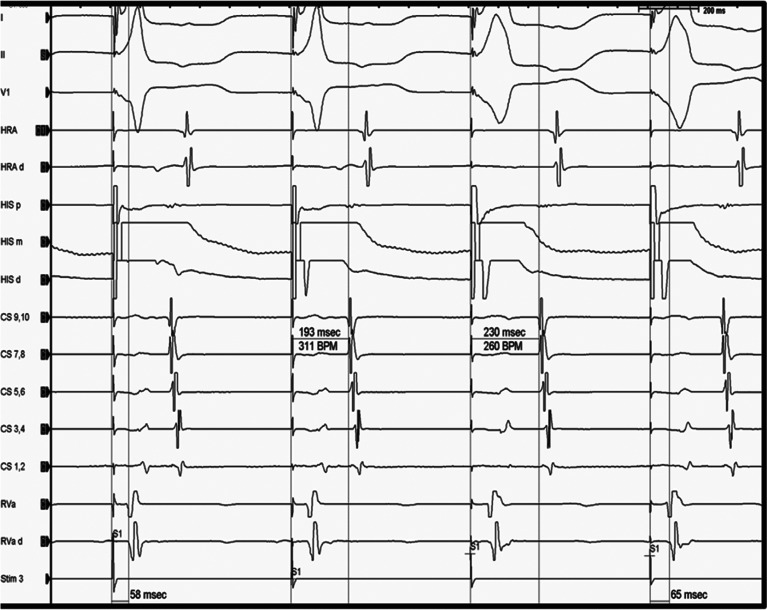
Para-Hisian pacing demonstrating the nodal response with the stim–A time during His capture being less than the stim–A time during the loss of His capture. Reproduced with permission from Kanjwal K, George A, Mainigi SK. Establishing the mechanism of supraventricular tachycardia in the electrophysiology laboratory. *J Innov Card Rhythm Manag*. 2012;4(4):1217–1230. CS: coronary sinus; d: distal; HRA: high right atrium; m: middle; p: proximal; RVa: right ventricular apex; Stim: stimulation.

**Figure 3:**
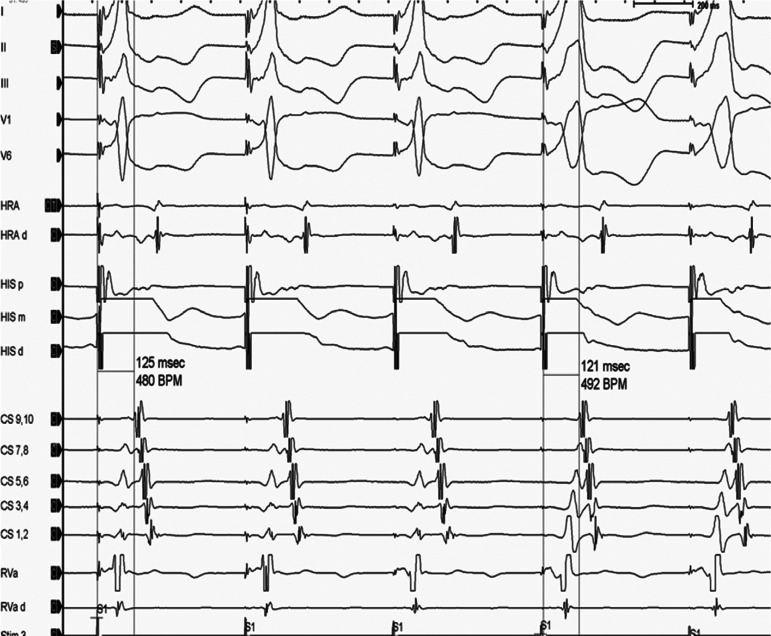
Demonstration of an extra-nodal response during para-Hisian pacing with similar stim–A time during His capture and the loss of His capture. Reproduced with permission from Kanjwal K, George A, Mainigi SK. Establishing the mechanism of supraventricular tachycardia in the electrophysiology laboratory. *J Innov Card Rhythm Manag*. 2012;4(4):1217–1230. CS: coronary sinus; d: distal; HRA: high right atrium; m: middle; p: proximal; RVa: right ventricular apex; Stim: stimulation.

The time from the stimulation artifact to the subsequent atrial signal is measured during His capture and during the loss of His capture. The expectation is that, in the setting of no accessory pathway, the loss of His capture will result in a widening of the QRS complex and a simultaneous increase in the stimulation–atrial (stim–A) time **(Figure 2)**. In contrast, the presence of a septal accessory pathway will result in identical stim–A times both with and without His capture **(Figure 3)**. During this maneuver, it is very important to make sure that there is no capture of the local atrium as this could cloud the results. The presence of a short stimulation to proximal coronary sinus atrium (< 60 ms) and stimulation to the high right atrium (< 70 ms) is suggestive of the direct capture of the atrium from the pacing catheter (**Figure 1**, first beat). A stim–A electrogram time of more than 90 ms in the proximal coronary sinus and 100 ms in the high right atrium argues strongly against direct atrial capture.^2^

In our patient, the first beat was likely a direct atrial capture as the stim–A time was short (< 60 ms), the second beat was a pure His capture, and the third beat was both His and ventricular capture. The last beat was a non-capture.

**Figure 4:**
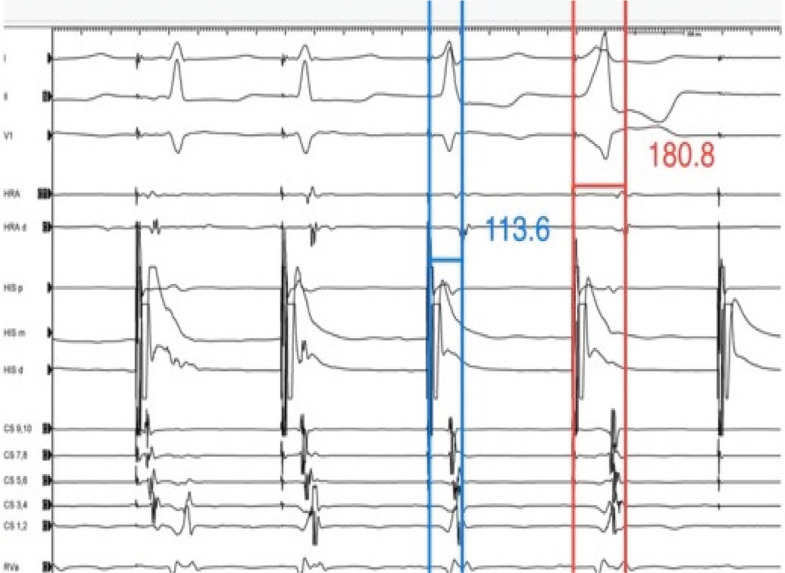
Demonstration of an extra-nodal response with the stim–A time during His capture being shorter than the stim–A time during the loss of His capture. However, during the loss of His capture (ventricular capture only), the activation changes and the retrograde conduction occur over the left-sided accessory pathway. CS: coronary sinus; d: distal; HRA: high right atrium; m: middle; p: proximal; RVa: right ventricular apex.

Based on the response seen in our patient **(Figures 1 and 4)**, it could be called a nodal response as the stim–A time during the loss of His capture was 180 ms and that during the His capture was 113 ms. However, a closer look at the fourth beat in **Figure 1** (ventricular capture) reveals an eccentric retrograde conduction over a left-sided accessory pathway. The patient was found to have a left posterior accessory pathway and underwent a successful ablation of the same.

There are several valuable teaching points illustrated in the **Figure 1** tracing. First, para-Hisian pacing only differentiates retrogradely conducting septal accessory pathways from retrograde conduction over the AV node. A “nodal” response can be seen in the presence of pathways away from the septum. A “nodal” response to a para-Hisian pacing can also be seen in pathways with a decremental retrograde conduction and pathways that conduct antegrade only.

Second, while performing the para-Hisian maneuver, close attention should be paid to the retrograde atrial activation sequence. Although the maneuver revealed a “nodal” response, close attention to the change in retrograde atrial activation during the loss of His capture (**Figure 1**, fourth beat) provided a clue toward the correct diagnosis in this patient.

Third, this tracing is unique as, in one snapshot (from beat 1 through beat 5) there are examples of a direct atrial capture, His capture, both His and ventricular capture, only ventricular capture, and loss of capture. The electrophysiology trainees should be able to understand the mechanism of each beat. Electrophysiologists need to be aware of various pitfalls of the maneuvers used during EP studies to accurately identify tachycardia mechanisms.
